# SQSTM1/p62 Interacts with HDAC6 and Regulates Deacetylase Activity

**DOI:** 10.1371/journal.pone.0076016

**Published:** 2013-09-27

**Authors:** Jin Yan, Michael Lamar Seibenhener, Luis Calderilla-Barbosa, Maria-Theresa Diaz-Meco, Jorge Moscat, Jianxiong Jiang, Marie W. Wooten, Michael C. Wooten

**Affiliations:** 1 Department of Biological Sciences, Cellular and Molecular Biosciences Program, Auburn University, Auburn, Alabama, United States of America; 2 Sanford-Burnham Medical Research Institute, La Jolla, California, United States of America; 3 Department of Pharmacology, Emory University School of Medicine, Atlanta, Georgia, United States of America; West Virginia University, United States of America

## Abstract

Protein aggregates can form in the cytoplasm of the cell and are accumulated at aggresomes localized to the microtubule organizing center (MTOC) where they are subsequently degraded by autophagy. In this process, aggregates are engulfed into autophagosomes which subsequently fuse with lysosomes for protein degradation. A member of the class II histone deacetylase family, histone deacetylase 6(HDAC6) has been shown to be involved in both aggresome formation and the fusion of autophagosomes with lysosomes making it an attractive target to regulate protein aggregation. The scaffolding protein sequestosome 1(SQSTM1)/p62 has also been shown to regulate accumulation and autophagic clearance of protein aggregates. Recent studies have revealed colocalization of HDAC6 and p62 to ubiquitinated mitochondria, as well as, ubiquitinated protein aggregates associated with the E3 ubiquitin ligase TRIM50. HDAC6 deacetylase activity is required for aggresome formation and can be regulated by protein interaction with HDAC6. Due to their colocalization at ubiquitinated protein aggregates, we sought to examine if p62 specifically interacted with HDAC6 and if so, if this interaction had any effect on HDAC6 activity and/or the physiological function of cortactin-F-actin assembly. We succeeded in identifying and mapping the direct interaction between HDAC6 and p62. We further show that this interaction regulates HDAC6 deacetylase activity. Data are presented demonstrating that the absence of p62 results in hyperactivation of HDAC6 and deacetylation of α-tubulin and cortactin. Further, upon induction of protein misfolding we show that p62 is required for perinuclear co-localization of cortactin-F-actin assemblies. Thus, our findings indicate that p62 plays a key role in regulating the recruitment of F-actin network assemblies to the MTOC, a critical cellular function that is required for successful autophagic clearance of protein aggregates.

## Introduction

Misfolded proteins are thought to be sequestered into aggregates for the protection of cells as accumulation of mis-functional proteins can be toxic [1]. This process was originally attributed to ubiquitin tagging of defective proteins leading to their recruitment into aggresomes that are degraded by autophagy(aggresome-autophagy pathway) [2]. However, recent advancements have shown that protein recruitment can also occur in an ubiquitin-independent manner [3]. The Class II histone deacetylase HDAC6has been associated with aggresome formation in both ubiquitin dependent [4], [5] and independent pathways [3]suggesting HDAC6 may play a pivotal role in both protein accumulation and cell protection. HDAC6 is predominantly localized to the cytoplasm, a feature that distinguishes it from other HDAC family members [6]. HDAC6 contains two catalytic domains, DD1 and DD2 [7], as well as, a C-terminal ubiquitin binding domain BUZ/ZnF-UBP [4], [8], [9]. Polyubiquitinated protein aggregates are recruited to HDAC6 through this BUZ domain [4], [10], while deacetylase activity is regulated by one or both of the internal catalytic domains [7], [11]. It has been proposed that HDAC6 facilitates loading of aggregated proteins onto the dynein motor protein complex by serving as an adaptor between ubiquitinated protein aggregates and dynein [4].As such, a functional interaction exists between HDAC6, the motor protein dynein, and polyubiquitinated proteins in aggresome formation at the microtubule organizing center (MTOC) [4].Knockdown of HDAC6 results in impairment of polyubiquitinated proteins recruitment to dynein and subsequent transport to the MTOC leading to an aggresome-deficient phenotype [4].Interestingly, the role of HDAC6 in aggresome-autophagy pathway is not solely that of an adaptor protein as deacetylation of its substrate cortactin is required for autophagosome-lysosome fusion [4], [12]. Thus, both accumulation of protein aggregates at aggresomes and their autophagic clearance occur in an HDAC6-dependent fashion.

A number of proteins have been found to regulate the activity of HDAC6. Both epidermal growth factor receptor(EGFR) [13] and casein kinase 2 (CK2) [14] regulate HDAC6 activity by phosphorylation, leading to changes in cellular acetylated tubulin levels. Expression of a CK2 phosphorylation site mutant of HDAC6(S458A) has been shown to abrogate recruitment of the HDAC6 substrate cortactin to aggresomes [14]. Failure of this recruitment leads to inability of the associated F-actin assembly network to organize properly which subsequently results in failure to clear aggregated proteins [12]. Other proteins, such as dysferlin, can also regulate HDAC6 deacetylation of tubulin by interfering with the interaction between HDAC6 and tubulin itself [15]. In addition, the HDAC6-interacting protein tau has been shown to inhibit HDAC6 deacetylase activity with overexpression of tau leading to inhibition of aggresome formation [16].

Interestingly, HDAC6 has recently been shown to also be involved in mito-aggresome formation that is associated with elimination of damaged mitochondria [17]. In this process, that closely resembles aggresome formation, the atypical protein kinase C (aPKC)-interacting protein sequestosome 1/p62 (hereafter referred to as simply p62) has been reported to co-localize with HDAC6 in ubiquitinated mito-aggresomes [17].p62 has been found to have myriad roles in cellular mechanics, not the least of which is a well-defined function in intracellular disposal pathways. In this role, p62 is involved in transport of both misfolded proteins and dysfunctional organelles to cellular degradation sites [18], [19]. This transport is accomplished via the microtubule network where the motor protein dynein “moves” cargoes along the microtubule concentrating damaged proteins and organelles into aggresomes or inclusion bodies [17], [20], [21]. Of particular importance in this role is the presence in p62 of a C-terminal UBA domain for binding of ubiquitin and ubiquitinated protein aggregates [18]. Recent studies have shown p62 is involved in inclusion body formation and selective autophagic clearance of ubiquitinated substrates [22], [23], [24]. In association with mitochondrial clearance by mitophagy, both p62 and HDAC6 are recruited to mitochondria ubiquitinated by parkin [17]. Similarly, both proteins have also been shown to interact with the E3 ubiquitin ligase TRIM50 [25] localizing to aggregate formation sites where they promote the sequestration and clearance of ubiquitinated proteins at aggresomes.

We have documented in previous work in our laboratory that loss of p62 abrogates movement of protein aggregates and organelles [18], [26]. Because both p62 and HDAC6 are known to be closely associated with aggregate clearance and both proteins show co-localization, we reasoned that p62 might directly or indirectly affect the activity of HDAC6.To test this hypothesis, we examined tubulin acetylation in a p62 knock-out model. Our goal was to determine what, if any, effect the presence of p62 has on the deacetylase activity of HDAC6 and how this might relate to our previous observation of impaired motor transport. In the results reported here, we identify a specific binding domain of p62 which does interacts with a catalytic domain of HDAC6 resulting in modulation of HDAC6 deacetylase activity. We show that lack of p62 hyper-activates HDAC6 resulting in elevated de-acetylation of the HDAC6 specific substrates α-tubulin and cortactin. We also reveal that elevation of HDAC6 activity by loss of p62 leads to an increased association of F-actin network assemblies with aggregates containing HDAC6 which are unable to move to the MTOC for autophagic degradation.

## Materials and Methods

### Cell Culture and Transfection

Human embryonic kidney (HEK) 293 cells from the American Type Culture Collection were grown as described previously [27]. Transfection was achieved using the Mammalian Cell Transfection Kit (EMD-Millipore, Billerica, MA). Wild Type (WT) and p62 knock-out (p62KO) mouse embryonic fibroblasts (MEF) were derived from E13.5 mouse embryos [28] and grown in DMEM supplemented with 10% Fetal Calf Serum and antibiotics at 37°C with high humidity and 5%CO_2_. Transfection of MEF cells was carried out using Lipofectamine 2000 (Life Technologies, Grand Island, NY).

### Antibodies and Reagents

Monoclonal antibodies for α-tubulin, acetylated α-tubulin and FLAG-tag were obtained from Sigma Chemical (St. Louis, MO). p62 monoclonal antibody was purchased from Abcam (Cambridge, MA). All other antibodies were purchased from Santa Cruz Biotechnology (Dallas, TX). All reagents were purchased from Sigma Chemical (St. Louis, MO). Tubacin and nil-Tubacin were a generous gift from Dr. Stuart Schreiber, Harvard University.

### Immunoprecipitation and Western Blot

Either HEK or MEF cells were lysed on ice using Triton Lysis Buffer (50 mM Tris-HCl, pH 7.5, 150 mM NaCl_2_, 10 mM NaF, 0.5% TX-100, 1 mM Na_3_VO_4_, 1 mM PMSF, 2 µg/ml aprotinin and leupeptin). Protein concentration was determined by Bradford Assay (Bio-Rad, Hercules, CA) prior to immunoprecipitation. Lysates were rotated overnight at 4°C with primary antibody followed by 3 hours with anti-IgG-agarose beads. Precipitates were washed with Triton Lysis Buffer a total of 3 times prior to the addition of 1X Sample Buffer. Samples were separated by SDS-PAGE and transferred to nitrocellulose for Western blotting.

### GST-Pulldown Assay

*E. coli* cells expressing GST-p62 were grown in 2xYT media (16 g tryptone, 10 g yeast extract, 5 g NaCl, 0.49 g sodium citrate, 6.27 g K_2_HPO_4_, 1.63 g KH_2_PO_4_per 1 liter, pH 7.6 supplemented with 100 mM MgSO_4_ and antibiotic) for 12 hours. Expression was induced with 1 mM IPTG for 4 hrs. Bacterial cells were lysed with NETN buffer (20 mM Tris, pH 8.0, 100 mM NaCl_2_, 1 mM EDTA, 0.1% NP40, 2 µg/ml leupeptin, 1 mM PMSF). GST-p62 was purified from bacterial lysates by binding to glutathione-agarose beads overnight at 4°C. Bound beads were then washed 5 times with NETN followed by resuspension in NETN. Protein concentration was determined by Bradford Assay.

HEK cell lysates expressing HA-HDAC6 were added to 10 µg GST-p62 beads and allowed to bind overnight at 4°C. Following incubation, beads were washed 3 times with NETN buffer and 1X Sample Buffer added. Pulldown samples were separated on SDS-PAGE followed by Western blot.

### HDAC6 Activity Assay

HDAC6 was purified from WT and p62KO MEF cells by immunoprecipitation. HDAC activity was then measured using the HDAC Colorimetric Activity Assay kit (Biovision, Milpitas, CA). Briefly, HDAC6 immunoprecipitates were incubated with a HDAC colorimetric substrate consisting of polypeptide chains with acetylated lysine side chains. Following incubation per the manufacturer’s instructions, the reaction was stopped by incubation with developer. Colorimetric detection was determined at 405 nm.

### Immunofluorescence Microscopy

WT and p62KO MEF cells were fixed in 4% paraformaldehyde for 1 hour. Cells were then washed with PBS prior to permeabilization with0.1% TX-100/PBS for 10 minutes. After permeabilization, cells were blocked in 3% milk/PBS at room temperature for 4 hours before adding primary antibodies in blocking solution. Fluorescently tagged secondary antibodies (Life Technologies, Grand Island, NY) were added in block for 2 hours at room temperature. Actin was stained using AF350-phalloidin (Life Technologies, Grand Island, NY). Colocalization of proteins was visualized using a Nikon A1/T1 confocal microscope and the Nikon Elements software.

### Statistical Analysis

Means, standard errors, Student’s *t* tests and Chi-squared tests were calculated manually [29]. Chi-squared statistical were calculated under the assumption of unequal group variances with one-tailed p-values. P-values less than 0.05 were considered significant.

## Results

### p62 affects acetylated tubulin levels in mouse embryonic fibroblasts

**Figure 1:**
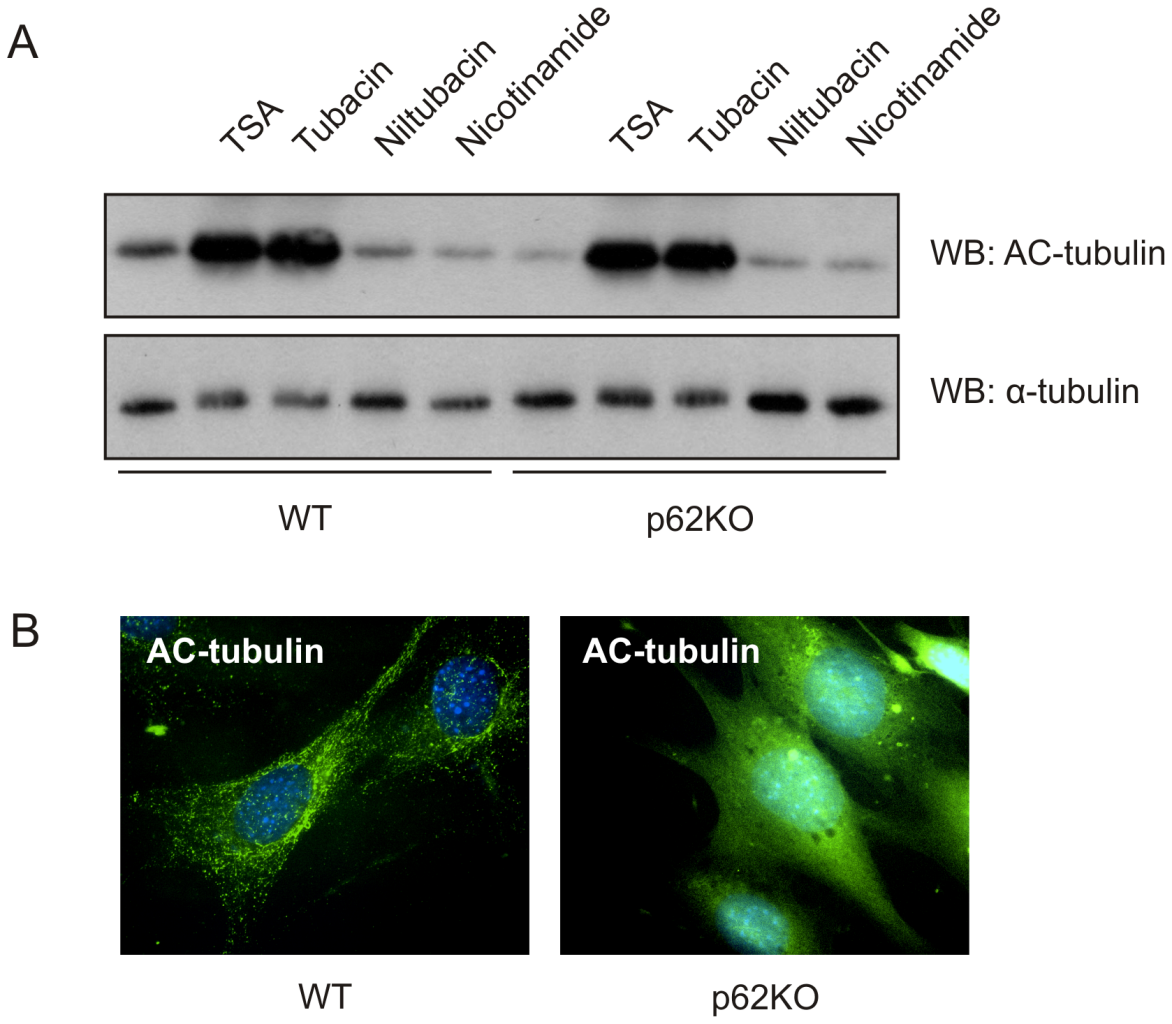
Absence of p62 leads to decreased acetylated-tubulin in mouse fibroblasts. (**A**) WT and p62KO MEF were treated with 2 µM TSA, 10 µM tubacin, 10 µM niltubacin, or 10 mM nicotinamide for 4 hours. Whole cell lysates were subjected to Western blot with anti-acetylated-α-tubulin and anti-α-tubulin antibodies. Blots were quantitated and relative expression values determined and analyzed for significance using Student's *t*-tests. [WT vs. p62KO Untreated: *t*(4) = 29.03, *p* = 4.19E-6; WT vs. p62KO TSA: *t*(4) = 0.27, *p* = 0.401; WT vs. p62KO Tubacin: *t*(4) = 0.49, *p* = 0.325; WT vs. p62KO Nil-tubacin: *t*(4) = 0.71, *p* = 0.259; WT vs. p62KO Nicotinimide: *t*(4) = 0.32, *p* = 0.382] (**B**) WT and p62KO MEF cells analyzed for acetylated α-tubulin by immunofluorescence.

Our first objective was to determine the activity level of HDAC6 in the absence of p62. For this experiment, we sought to examine tubulin acetylation levels in WT and p62KO MEF cells via Western blot with acetyl-tubulin specific antibody. While α-tubulin levels remained relatively constant between WT and p62KO cells, we observed a significant decrease in acetylation of tubulin in the absence of p62 (WT vs. p62KO: t(4) = 29.03, p = 4.19E-6) (Fig. 1A). Deacetylation of tubulin can be accomplished by the HDAC family of deacetylases [30], [31]. Upon treatment with the general class I and II HDAC inhibitor Trichostatin A (TSA), deacetylation of tubulin was inhibited while treatment with nicotinamide, a class III NAD+- dependent SIRT family inhibitor, did not inhibit deacetylation of tubulin. α-Tubulin is a specific substrate of HDAC6 [31], [32], [33]. In WT or p62KO cells, treated with the HDAC6 specific inhibitor tubacin, deacetylation of tubulin was inhibited while treatment with the inactive analog, nil-tubacin produced no evidence of deacetylation inhibition. Examination of the ultra-structure of tubulin filaments using immunofluorescence-based microscopy showed characteristic acetylated tubulin staining along filaments in WT cells but disorganized and diffuse acetyl-tubulin staining in cells lacking p62 (Fig. 1B). We concluded from these results that p62 could play a regulatory role in the deacetylase activity of the HDAC family member HDAC6.

### p62 interacts with the HDAC family member HDAC6

**Figure 2:**
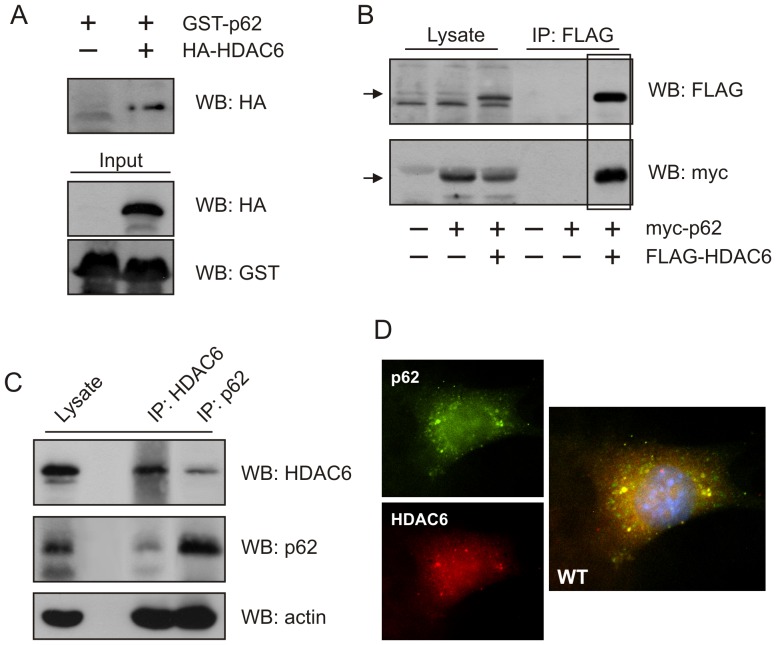
p62 specifically interacts with HDAC6. (**A**) Lysates of HEK cells transfected or not with HA-HDAC6 were subjected to GST-p62 pulldown assay. Tagged constructs were captured on glutathione sepharose beads and analyzed by Western blot with anti-HA and anti-GST antibodies. (**B**) HEK cells were transfected with FLAG-HDAC6 and myc-p62 constructs. HDAC6 was immunoprecipitated with FLAG-tag antibody and presence of co-precipitating myc-p62 (included in highlighted box) analyzed by Western blot with anti-myc antibody. Presence of transfected constructs were confirmed in the whole cell lysate. (**C**) Endogenously expressed p62 and HDAC6 was examined in WT MEF cells by immunoprecipitation with p62 or HDAC6 antibodies and analyzed by Western blot with corresponding antibodies. (**D**) Endogenous HDAC6 and p62 localization was examined by immunofluorescence in WT MEF cells.

In general, regulatory proteins interact directly with their substrates. To date, a number of proteins have been identified that regulate HDAC6 activity through physical interaction [15], [16], [34]. Thus, we hypothesized that the regulatory effect of p62 on the deacetylation activity of HDAC6 could be accomplished by direct interaction between p62 and HDAC6. Using bacterially expressed GST-tagged p62, a pulldown experiment incorporating exogenous HA-tagged HDAC6 protein was performed. Results from this experiment provided strong evidence of a direct interaction between p62 and HDAC6 in an *in vitro* environment (Fig. 2A). Interaction between the two proteins was further confirmed by co-precipitation using exogenously expressed constructs (Fig. 2B), as well as, endogenously expressed proteins (Fig. 2C) indicating interaction between p62 and HDAC6 occurred *in vivo* as well as *in vitro*. Evidence of endogenous p62 and HDAC6 co-localization was also observed when WT MEF cells were examined by immunofluorescence staining and confocal microscopy (Fig. 2D).

### Mapping the interaction sites between p62 and HDAC6

**Figure 3:**
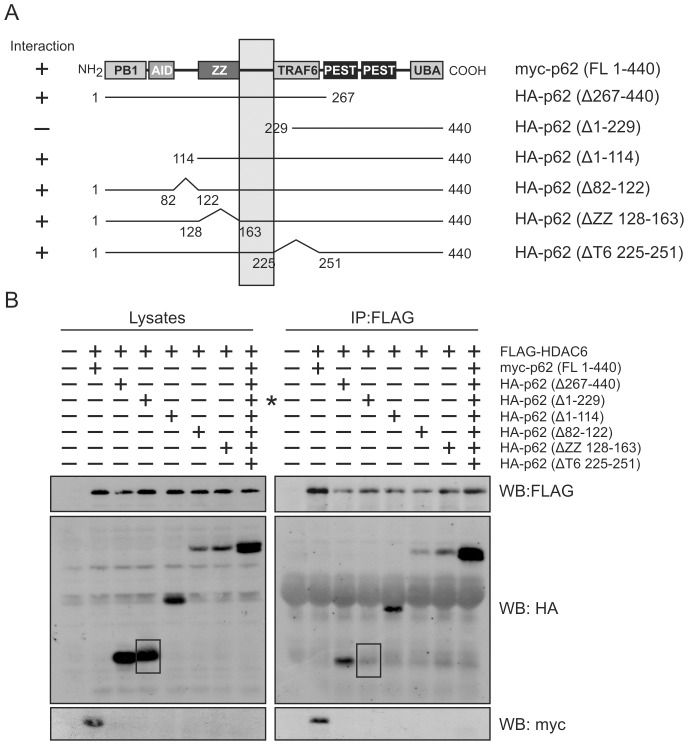
Mapping the interaction region on p62. (**A**) Schematic diagram of HA-tagged p62 deletion constructs. (**B**) HEK cells were transfected with full length FLAG-HDAC6 and HA-tagged p62 deletion mutants constructs. Full length p62 was myc-tagged. HDAC6 was captured with anti-FLAG antibody and co-precipitating myc-tagged full length p62 or HA-tagged p62 deletion constructs analyzed by Western blot with anti-HA antibody.

As a highly specific interaction was observed between p62 and HDAC6 (Fig. 2A-D), we next sought to determine the interaction domains of both proteins using deletion constructs. A full length tagged-p62 construct along with various internal domain deletions (Fig. 3A) were expressed in HEK cells with FLAG-HDAC6. Co-immunoprecipitation was performed using a FLAG-antibody for HDAC6 and Western blots were generated to the tagged-p62 deletion constructs. p62 interaction was seen for all constructs with the exception of the p62 (Δ1-229) which encompassed only the carboxyl terminal half of the protein (Fig. 3B). When alignment of all constructs was performed, the HDAC6 binding region was localized to residues 164–225, the undefined region between p62’s ZZ domain and the identified TRAF6 binding region (shaded area of Fig. 3A).

**Figure 4:**
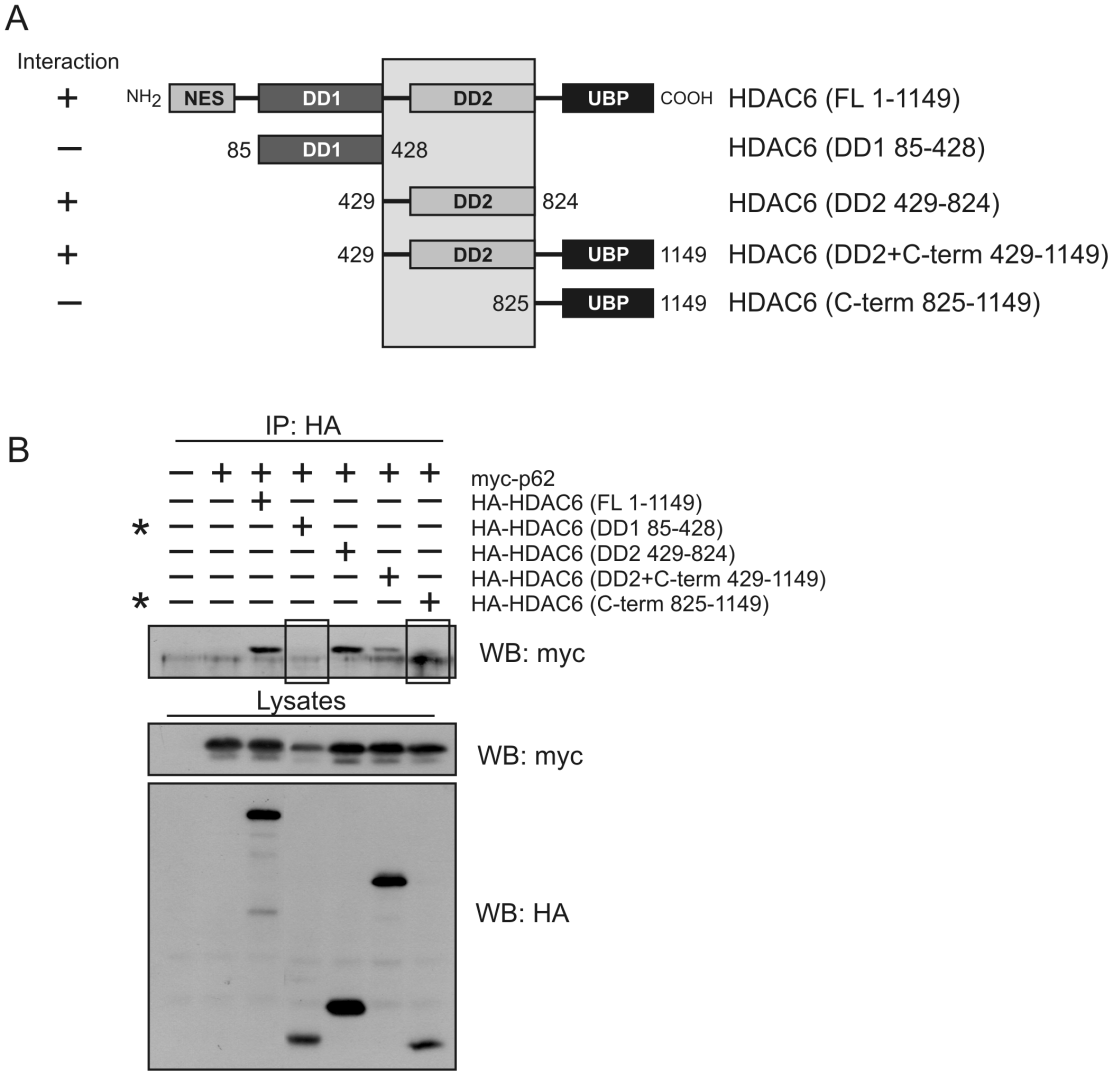
Mapping the interaction region on HDAC6. (**A**) Schematic diagram of HA-tagged HDAC6 truncation constructs. (**B**) HEK cells were transfected with full length myc-p62 and HA-tagged HDAC6 deletion constructs. p62 was captured with anti-myc antibody and co-precipitating HA-tagged HDAC6 constructs analyzed by Western blot with anti-HA antibody.

To map the region of HDAC6 interacting with p62, tagged HDAC6 truncation constructs (Fig 4A) were expressed in HEK cells along with myc-tagged full length p62 and co-immunoprecipitation performed as above. HDAC6’s catalytic domains DD1 and DD2 have been shown to play critical roles in the protein’s catalytic activity [11]. In particular, DD2 was indicated to specifically regulate the deacetylase activity of HDAC6 and to possess a catalytic site inhibited by tubacin [33]. Of the truncated HDAC6 constructs examined, only those containing the DD2 domain showed interaction with p62 (Fig.4B). When alignment of the constructs was performed, the p62 binding domain was localized to residues 429–824 which encompass the entirety of the DD2 domain (shaded area of Fig. 4A). Collectively, these experiments confirm the direct interaction between p62 and HDAC6 and map specific interaction domain within both proteins.

### p62 inhibits the deacetylase activity of HDAC6

**Figure 5:**
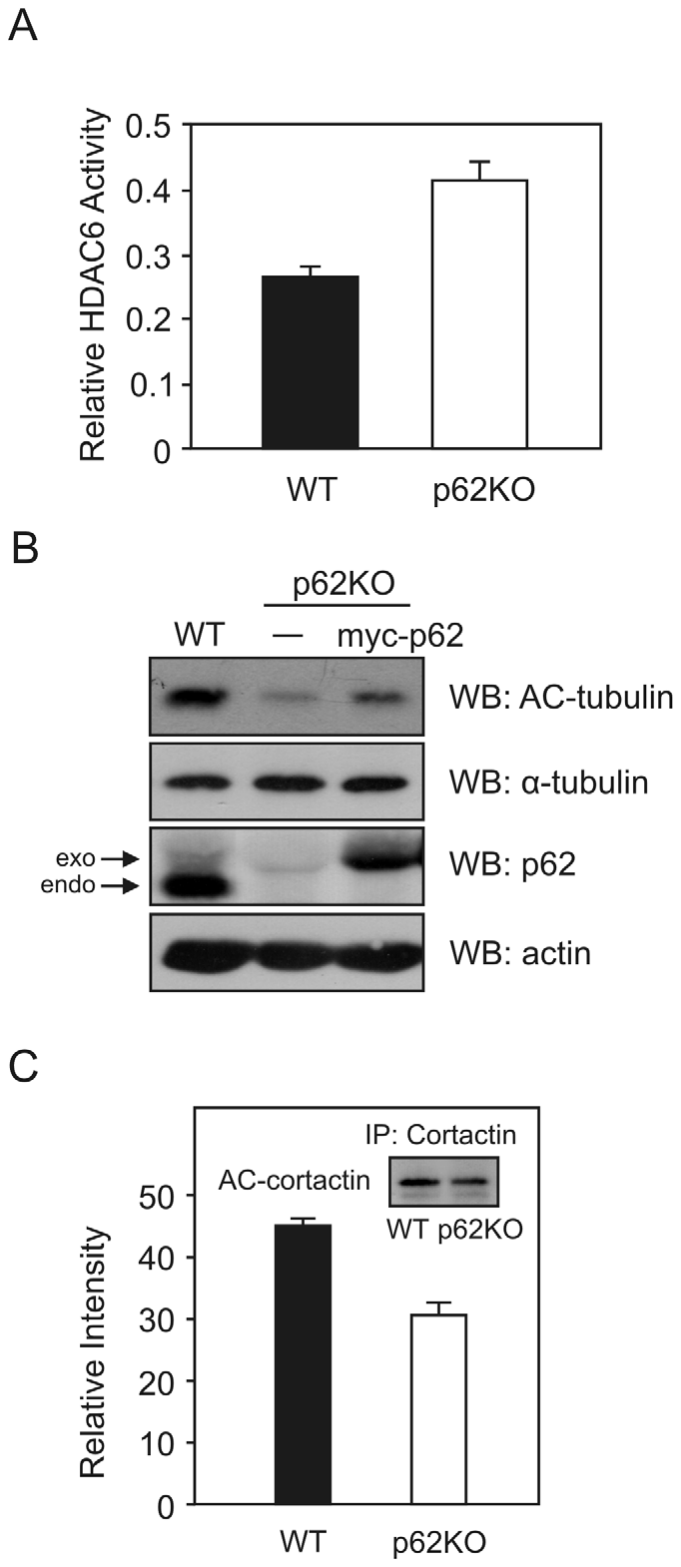
Absence of p62 enhances HDAC6 deacetylase activity. (**A**) Endogenous HDAC6 was immunoprecipitated with anti-HDAC6 antibody from WT and p62KO MEF cell lysates. HDAC6 immune complexes were incubated with acetylated lysine substrate. HDAC6 specific activity was measured colorimetrically with a spectrophotometer. Relative HDAC6 deacetylation activity is presented from three separate experiments (one-tailed *t* = 4.47; *p*<0.01). (**B**) myc-tagged full length p62 construct was transfected or not into p62KO MEF cells. Whole cell lysates were subjected to Western blot with anti-acetylated (AC)-α-tubulin, anti-α-tubulin, and anti-p62 antibodies. WT MEF cell lysates were loaded as control expression levels. Actin immuno-reactivity was included as a loading control and exogenous (exo) and endogenous (endo) bands of p62 are indicated. (**C**) Endogenous cortactin was immunoprecipitated from WT and p62KO MEF cells using anti-cortactin antibody and subjected to Western blot with anti-acetylated-cortactin. Relative acetylated cortactin intensity in WT and p62KO cells was calculated by normalizing acetylated cortactin over total cortactin on immunoprecipitates. Results are representative of three separate experiments (one-tailed *t* = 6.80; *p*<0.05).

We have shown evidence for a specific interaction between p62 and HDAC6 that correlates with increased HDAC6 specific deacetylation of an *in vivo* substrate, α-tubulin, in the absence of p62. Based on these results, we next sought to examine if the increase in HDAC6 activity was specific to α-tubulin or a generalized catalytic increase caused by p62 driven deregulation. HDAC6 was immunoprecipitated from WT and p62KO MEF cells and activity of the immune complex was determined by *in vitro* deacetylation of a commercial acetylated substrate (Fig. 5A). HDAC6 immunoprecipitated from p62KO cells showed significantly increased deacetylase activity using this *in vitro* substrate. As p62 does appear to regulate HDAC6 deacetylase activity on both *in vivo* and *in vitro* substrates, we reasoned that return of p62 to the p62KO null background would reestablish control of HDAC6 activity. Exogenous p62 was transfected into p62KO MEF cells and tubulin acetylation examined by Western blot (Fig. 5B). Acetylated tubulin levels were increased in transfected cells and, while not completely reaching WT acetylation thresholds, reestablishment of p62 regulated the specific HDAC6 deacetylation of tubulin.

### p62 is required for the physiological function of the cortactin-F-Actin assembly

As p62 was found to regulate HDAC6 activity on both *in vitro* and *in vivo* substrates, we next sought to examine its effect on HDAC6 activity in a physiological process. HDAC6 has been shown to be integral for the recruitment of cortactin [35], along with F-actin assemblies to perinuclear protein aggregates [12]. Once recruited, cortactin is deacetylated by active HDAC6 leading to autophagosome-lysosome fusion and protein aggregate clearance [12]. We reasoned that if HDAC6 activity is negatively regulated by the presence p62, cortactin acetylation levels in p62KO cells could be decreased. When cortactin was immunoprecipitated from either WT or p62KO MEF cells, acetylated cortactin levels were significantly decreased in the absence of p62 (Fig. 5C) further supporting a regulatory role for p62 in the activity of HDAC6.

**Figure 6:**
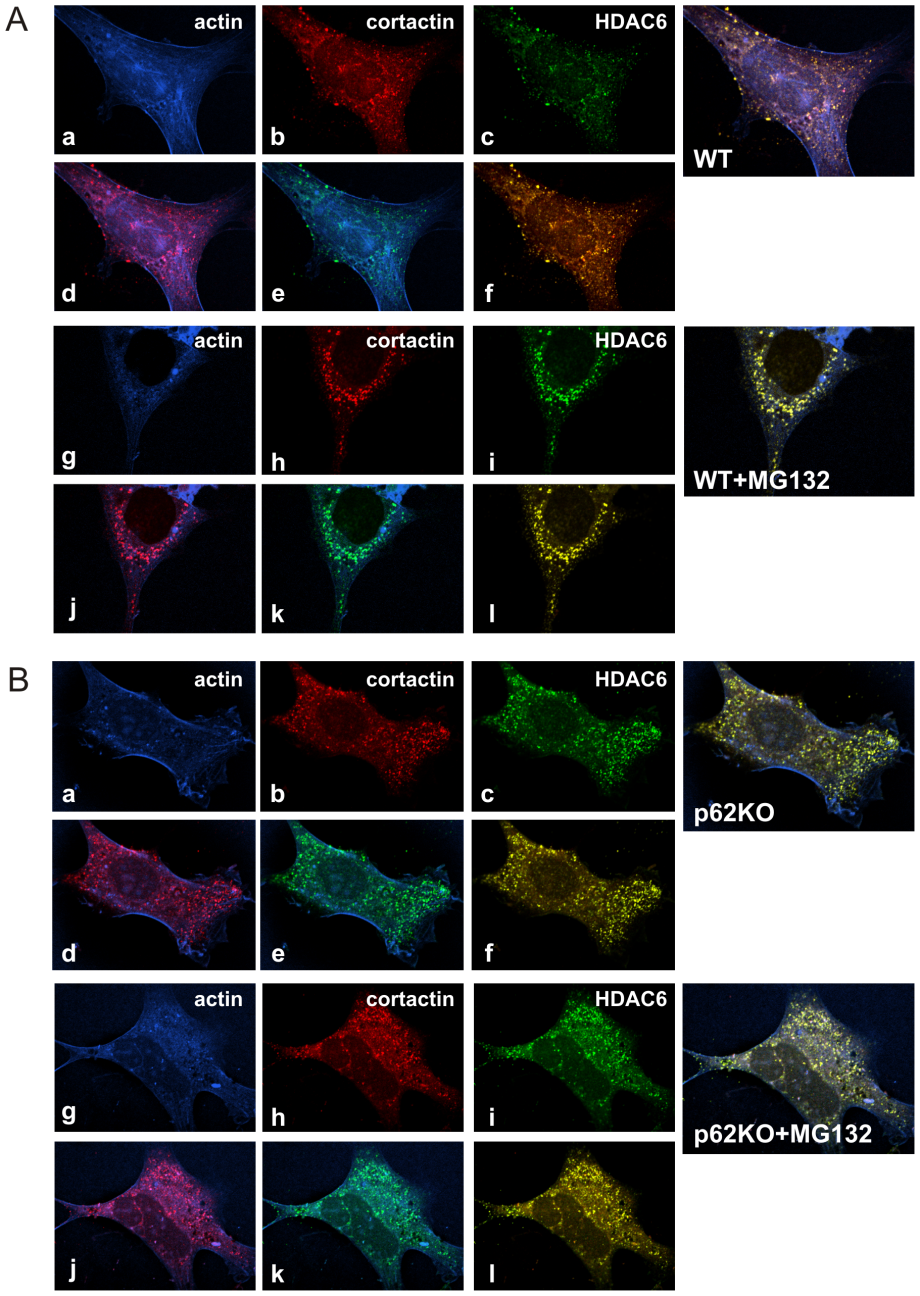
p62 regulates physiological function of cortactin-F-actin assembly. (**A**) WT MEF cells, either untreated (a-f) or treated (g-l),with 5 µM MG132 for 6 hours were fixed with paraformaldehyde. F-actin was stained with phalloidin-AF350. Endogenous cortactin was immuno-stained with Texas Red secondary antibody and endogenous HDAC6 immuno-stained with Oregon Green secondary antibody. Cells were analyzed with confocal microscopy. Panel identification is as follows: a and g – actin; b and h – cortactin; c and i – HDAC6; d and j – actin/cortactin; e and k – actin/HDAC6; f and l – cortactin/HDAC6. A fully merged image is shown to the right of each set of panels. (**B**) p62KO MEF cells, either untreated (a-f) or treated (g-l), with 5 µM MG132 for 6 hours, were subjected to the same immunofluorescence procedure as described above.

F-actin remodeling is required for quality control (QC) autophagy-dependent degradation of protein aggregates and deacetylation of cortactin is critical to this process [12]. In HDAC6 KO MEF cells, loss of HDAC6 prevents the co-localization of cortactin/F-actin with protein aggregates in an activity dependent fashion [12]. As we have shown that p62 plays a role in regulating HDAC6 activity on cortactin deacetylation, we sought to determine what role, if any, p62 plays in cortactin/F-actin assembly. We examined cortactin/HDAC6 colocalization at sites of F-actin assemblies in WT and p62KO MEF cells by immunofluorescence (Fig. 6) and quantitatively estimated Mander’s Overlap Coefficient values of colocalization [36] using the NIS Elements software (Nikon). Evidence of recruitment of cortactin to F-actin was seen in WT and p62KO cells as noted by colocalization of actin and cortactin (Fig.6A d – Mander’s  = 0.877; Fig. 6B d – Mander’s  = 0.729). However, co-localization of HDAC6 and cortactin was more evident in p62KO MEFS (Fig. 6B f – Mander’s  = 0.968) compared to WT (Fig. 6A f – Mander’s  = 0.864) as would be expected if HDAC6 activity is deregulated by the absence of p62. If this is indeed the case, increased HDAC6 activity would cause deacetylation of cortactin leading to increased cortactin/F-actin association with aggregates (Fig. 6A vs Fig. 6B merge for non-treated cells).

To induce QC-autophagy, cells were treated with MG132 to promote protein misfolding. We observed an overall increase in HDAC6-cortactin co-localization in the induced samples for WT (Fig. 6A f – Mander’s  = 0.864 vs. Fig. 6A l – Mander’s  = 0.987). However, untreated p62KO cells showed high levels of co-localization between HDAC6 and cortactin that were unresponsive to MG132 treatment (Fig. 6B f – Mander’s  = 0.968 vs. Fig. 6B l – Mander’s  = 0.971). This result is indicative of higher *in vivo* stress levels due to the lack of p62 in these cells [37]. Importantly, induced HDAC6-cortactin/F-actin assemblies in WT cells were predominately found in the perinuclear region of the cell as would be expected in a normally functioning cell. Conversely, these assemblies remained localized in the cytosol in the absence of p62 (Fig. 6A vs 6B, merge for MG132-treated cells). Therefore, we concluded that while recruitment of cortactin to F-actin is unaffected in the absence of p62 (Fig. 6A k; Fig. 6B k), F-actin remodeling is abrogated by the lack of p62 (Fig. 6B merge for MG132-treated cells).

## Discussion

HDAC6 is one of the most extensively studied members of the histone deacetylase family of proteins [38]. HDAC6 is exclusively localized in the cell cytoplasm and has a number of cytoplasmic substrates including α-tubulin [7], cortactin[35], HSP90 [39] and peroxiredoxin [40]. Cytoplasmic localization and the functions of its substrates implicate HDAC6 in a number of cellular regulatory processes. Because of its involvement in multiple activities, how HDAC6 is regulated has become an area of intense interest.

As is true for many essential proteins, HDAC6 appears to be regulated at multiple levels. One observed mode of HDAC6 regulation is specifically associated with a change in its localization within the cytoplasm. HDAC6 can be translocated by association with its substrate HSP90, along with Rac1, to membrane ruffles after PDGF stimulation where it can influence actin dynamics [41] resulting in cell migration. Another mode of HDAC6 activity regulation is via post translational modification such as phosphorylation. HDAC6 is phosphorylated by the EGFR after ligand-induced receptor binding leading to increased acetylated tubulin and delivery of endocytosed EFGR to the lysosome for degradation [13]. GSK3β-dependent phosphorylation may also enhance the activity of HDAC6 leading to decreased tubulin acetylation and an inhibition in mitochondrial motility [42].

Yet a third method of HDAC6 regulation is the direct or indirect binding of various partners to HDAC6 itself. A complex composed of HDAC6, farnesyltransferase and microtubules can be essential for HDAC6 activity. Disruption of this complex by inhibition of the transferase activity results in increased tubulin acetylation [43]. Similarly, by direct binding to tau, the deacetylase activity of HDAC6 is inhibited resulting in increased tubulin acetylation [16]. An excess of tau protein may act as an HDAC6 inhibitor preventing autophagy induced by proteasome inhibition. Thus, tau has been shown to not only directly inhibit the deacetylase activity of HDAC6, but also impair HDAC6-dependent autophagy [16].

We have shown here that the scaffolding protein p62 can directly bind to one of the two catalytic domains of HDAC6 and that p62 can regulate the deacetylase activity of HDAC6. As the binding site for direct p62 interaction with HDAC6 is located in the catalytic DD2 domain, it is possible that this direct interaction is responsible for HDAC6 inhibition. As HDAC6 requires both DD1 and DD2 catalytic domains to function [11], physical interaction of p62 with one of these domains could inhibit HDAC6 deacetylase activity. However, p62 was first recognized as an aPKC-interacting protein where it supports the phosphorylation activity of aPKC [44]. It is therefore possible that the interaction of p62 with HDAC6 provides a scaffold for the recruitment of aPKC. As discussed above, phosphorylation can lead to change of HDAC6 activity [13], [42]. Once present, aPKC could phosphorylate and alter HDAC6 activity. In fact, we have shown that HDAC6, p62 and aPKC do exist in a ternary complex (data not shown). However, the physiological role of aPKC on HDAC6 phosphorylation and subsequent activation has not been elucidated. Our data did clearly show that a specific and direct interaction between p62 and HDAC6 regulates the deacetylase activity of HDAC6 towards its substrates.

α-tubulin together with β-tubulin form the heterodimeric building blocks of microtubules [45]. Acetylation of α-tubulin at Lys40 is thought to stabilize microtubules in the cell [46], [47]. This line of thought is somewhat controversial as to whether acetylation is the cause or consequence of stabilization [48]. Regardless, acetylation of α-tubulin is a characteristic of stable microtubules [49], [50] along with tau interaction [51]. Destabilization of microtubules by deacetylation or drug treatment has been linked to neurodegenerative diseases [52]. We have previously shown that p62KO mice display characteristics biochemically and behaviorally similar to an Alzheimer’s disease mouse model [37]. Thus, dysregulation of HDAC6 activity by loss of p62 could affect the stability of microtubules leading to neurodegenerative disease.

In addition to its role in stabilization of microtubules, tubulin acetylation also plays a role in enhancing protein trafficking along microtubules in polarized cells [53]. Tubulin acetylation enhances recruitment of the molecular motors kinesin and dynein to microtubules to promote vesicular transport [53], [54]. In fact, kinesin binding and transport of the cargo protein Jip-1 [53], as well as kinesin bound mitochondria in neurons [42],are enhanced by tubulin acetylation. Increased HDAC6 activity could disrupt trafficking in the cell by removing acetyl groups from α-tubulin implicating p62 not only in the formation of protein aggregates but also in the regulation of their transport to the processing centers of the cell.

HDAC6 has emerged as an important player in the regulation of cellular protein aggregates due to its cytoplasmic localization, its association with the microtubule transport network and its ability to associate with ubiquitinated substrates via its C-terminal BUZ/ZnF-UBP domain [4], [8], [9]. HDAC6 has been recognized as a main regulatory component of the aggresome, the MTOC localized inclusion body where excess protein aggregates are trafficked and disposed [4]. HDAC6 has also been implicated in removal of aggresomes by QC-autophagy linking HDAC6 to this cellular clearance process as well [5], [12], [55]. HDAC6 is part of the F-actin remodeling machinery where it facilitates the fusion of autophagosomes with lysosomes leading to autophagic clearance [12]. HDAC6 deacetylates cortactin as part of this complex, promoting F-actin remodeling leading to fusion of the autophagosome to lysosomes and protein aggregate clearance at the aggresome [12].

p62 is also implicated in aggregate clearance by its ability to bind to both the autophagic marker protein LC3 and ubiquitinated substrates via its C-terminal UBA domain leading to protein aggregate clearance [22], [23]. p62 has been suggested as a shuttle protein to transport ubiquitinated substrates directly to the autophagosome [22]. Recently, p62 has also been implicated in the autophagic clearance of non-ubiquitinated STAT5A_ΔE18 as well [3]. In this study, knockdown of p62 inhibited LC3 lipidation and autophagosome formation, however accumulation of non-ubiquitinated STAT5A_ΔE18 was not observed suggesting incomplete inhibition of autophagy in p62 knockdown cells. Adapter proteins, such as Nbr1, have been shown to be recruited to ubiquitin aggregates where they are thought to play a role in autophagy similar to p62 to shuttle tagged proteins for autophagic clearance [56]. Thus, even with knockdown of p62, some level of autophagy persists.

Our data expands on the model where hyperactivated HDAC6 results in hypoacetylation of cortactin leading to a dramatic increase in recruitment of cortactin to F-actin (Fig. 6A e vs. Fig. 6B e). Interestingly, our data demonstrated that absence of p62 resulted in increased colocalization of cortactin-F-actin assemblies with aggregates containing HDAC6 during QC-autophagy (Fig. 6A vs. Fig. 6B merge for MG132-treated cells). However, perinuclear aggresome-like aggregates containing HDAC6 were seen to colocalize with cortactin/F-actin assemblies in WT cells while they accumulate in the cytoplasm of p62KO cells. It is possible that under basal conditions, the level of compensatory clearance pathways, such as Nbr1, did not show sufficient clearance of aggregates by our detection methods. However, when QC-autophagy was stimulated by protein misfolding, we would expect to see some clearance of aggregated proteins to the MTOC even in the absence of p62. This was not the case as the localization of cortactin-F-actin assemblies remained localized to the cytoplasm in p62KO cells (Fig. 6B j).

Both HDAC6 and p62 are suggested to play independent roles in aggregate clearance [2]. p62 is thought to be responsible for transporting aggregated proteins to the autophagosome [22] while HDAC6 is shown to be responsible for autophagosome-lysosome fusion [12]. Both proteins are implicated in transport of aggregated proteins to the aggresome for disposal [2]. Based on our observation of direct interaction between the two proteins, we propose that p62 likely plays a dual role in autophagy, not only in the formation of the autophagosome, but also in the regulation of HDAC6 activity which is responsible for autophagosome-lysosome fusion. At basal levels, the ratio of p62 and HDAC6 maintain homeostasis of the autophagic process. However, absence of p62 leaves HDAC6 in a hyperactive state, resulting in excessive cortactin deacetylation and F-actin network assembly around aggregates (Fig. 6B e; Fig 6B k).

It is interesting to also postulate on the effects of hypoacetylation on α-tubulin. Acetylation of microtubules, comprised of both α and β-tubulin, is suggested to alter the structure and dynamics of microtubules [57], [58]. Our data show that F-actin assemblies surrounding proteins aggregates remained in the cytoplasm in the absence of p62. While this could be due to a requirement for p62 for a direct attachment to motor proteins along the microtubule for retrograde transport to the MTOC, no such interaction between p62 and dynein has been shown. It is intriguing to suggest the deacetylase activity of HDAC6 affects the movement of motor proteins along the microtubule. Under the control of p62 associating with HDAC6, tubulin acetylation could be regulated. However, if unregulated by the absence of p62, motor protein transport could be compromised which would prevent aggregate movement and aggresome formation. This is indeed the case for what is seen in the absence of p62 with cytoplasmic localization of cortactin-F-actin assemblies upon induction of protein misfolding. More investigation into the role of acetylation on microtubule dynamics is warranted to shed further light on this process.

Reversible protein acetylation is being recognized in a wide range of cellular processes as a regulatory mechanism. The functional significance of acetylation/deacetylation has in the past been relegated to our understanding of transcriptional control process in the nucleus of the cell [38]. However, cytoplasmic acetylation/deacetylation of proteins is becoming more recognized as a regulatory event and HDAC6 is at the crux of cytoplasmic deacetylation. Our results support a central role for p62 in the regulation of HDAC6 deacetylase activity.
